# Inflammation-related citrullination of matrisome proteins in human cancer

**DOI:** 10.3389/fonc.2022.1035188

**Published:** 2022-12-01

**Authors:** Pekka Rappu, Ujjwal Suwal, Elina Siljamäki, Jyrki Heino

**Affiliations:** Department of Life Technologies and InFLAMES Research Flagship Center, University of Turku, Turku, Finland

**Keywords:** citrullination, inflammation, extracellular matrix, cancer, proteomics

## Abstract

**Introduction:**

Protein arginine deiminases (PADs) are intracellular enzymes that may, especially in pathological conditions, also citrullinate extracellular substrates, including matrisome proteins such as structural proteins in extracellular matrix (ECM). PADs are abundantly expressed in human cancer cells. Citrullination of matrisome proteins has been reported in colon cancer but the phenomenon has never been systematically studied.

**Methods:**

To gain a broader view of citrullination of matrisome proteins in cancer, we analyzed cancer proteomics data sets in 3 public databases for citrullinated matrisome proteins. In addition, we used three-dimensional cell cocultures of fibroblasts and cancer cells and analyzed citrullination of ECM.

**Results and discussion:**

Our new analysis indicate that citrullination of ECM occurs in human cancer, and there is a significant variation between tumors. Most frequently citrullinated proteins included fibrinogen and fibronectin, which are typically citrullinated in rheumatoid inflammation. We also detected correlation between immune cell marker proteins, matrix metalloproteinases and ECM citrullination, which suggests that in cancer, citrullination of matrisome proteins is predominantly an inflammation-related phenomenon. This was further supported by our analysis of three-dimensional spheroid co-cultures of nine human cancer cell lines and fibroblasts by mass spectrometry, which gave no evidence that cancer cells or fibroblasts could citrullinate matrisome proteins in tumor stroma. It also appears that in the spheroid cultures, matrisome proteins are protected from citrullination.

## Introduction

Protein arginine deiminases 2 and 4 (PAD2 and PAD4) are abundant calcium-dependent enzymes in cancer cells and tissues (1, 2). In addition, PAD1 has been shown to be overexpressed in triple-negative breast cancer (3). In murine model the inhibition of PADs reduced liver metastasis formation by colon cancer cells (4). However, in human colorectal carcinoma high expression levels of nuclear PAD4 and cytoplasmic PAD2 and PAD4 have been associated with an increase in overall survival (5). PAD2 and PAD4 both catalyze the deimination of protein associated arginine residues, *i.e.*, they citrullinate proteins (6). PADs are typically intracellular enzymes that regulate the function of proteins such as chromatin forming histones in the nucleus and modify cellular signaling in the cytosol (1, 2).

Protein citrullination has been intensively studied in chronic inflammation, especially in rheumatoid arthritis (7–9). Many inflammatory cells, including neutrophils, monocytes and macrophages, express PADs and the enzymes are released into extracellular space with an incompletely known mechanism. Citrullination of extracellular matrix (ECM) molecules, especially collagens, creates new antigenic epitopes and promotes autoimmunological responses that may play a critical role in the pathogenesis of rheumatoid disease (7–9). Furthermore, the deimination of arginine residues in ECM proteins, such as collagens, fibronectin and matrix metalloproteinases, may also affect their function as mediators of cell adhesion and matrix remodeling (10–12). The latent preform of transforming growth factor β (TGF-β) is bound to ECM and is activated by an arginine-glycine-aspartic acid motif (RGD motif) and in an integrin adhesion receptor-dependent manner. Citrullination of the arginine residue in the RGD motif can also prevent TGF-β activation (13). Citrullination of yet another ECM protein, namely fibulin-5 (FBLN5), by PAD2 has been detected in lungs of young mice where the process promotes elastogenesis (14). Genetic ablation of PAD2 results in attenuated elastogenesis *in vitro* and age-dependent emphysema *in vivo* (14).

Whereas citrullination has been studied extensively in rheumatoid arthritis (15), citrullination of matrisome proteins in cancer has not been systematically analyzed. Matrisome contains both ECM and ECM-associated proteins (16). One previous report indicates that citrullination of matrisome may have important role during the growth of liver metastasis in colorectal cancer (4). Furthermore, protein citrullination may also be an important source of neoantigens in cancer (17).

We have analyzed cancer proteomics data sets in public proteomics databases PRIDE, MassIVE and CPTAC for citrullinated ECM proteins. We show that citrullination of ECM occurs in human cancer with a significant variation between tumors. We also report a correlation between immune cell marker proteins and matrisome citrullination in human tumors, suggesting that ECM citrullination is predominantly an inflammation-related phenomenon in cancer. This is further supported by our experiments utilizing spheroid cell cultures of cancer cell lines and fibroblasts, which gave no evidence that cancer cells or fibroblasts could citrullinate ECM proteins.

## Materials and methods

### Cell lines and cell culture

UT-SCC-7 (RRID : CVCL_7868) cell line was established from surgically removed metastatic cutaneous squamous cell carcinoma (cSCC) of the skin in Turku University Hospital, and human head and neck SCC cell line UT-SCC-2 (RRID : CVCL_7820) was established from primary SCC of the oral cavity in Turku University Hospital (18, 19). UT-SCC-7, UT-SCC-2 and HepG2 (RRID : CVCL_0027, hepatocellular carcinoma) cells were a kind gift from Professor Veli-Matti Kähäri (Department of Dermatology, University of Turku and Turku University Hospital, Turku, Finland). A549 (RRID: CVCL_0023, lung carcinoma), MDA-MB-231 (RRID : CVCL_0062) and MCF7 (RRID : CVCL_0031) (breast adenocarcinomas) cells were purchased from ATCC. SKOV3 (RRID : CVCL_0532) and Caov-3 (RRID : CVCL_0201) (ovarian adenocarcinomas) cell lines were a kind gift from Professor Klaus Elenius (Institute of Biomedicine, University of Turku, Finland). Caco-2 (RRID : CVCL_0025, colorectal adenocarcinoma) cells were kindly provided by Professor Diana Toivola (Faculty of Science and Engineering, Åbo Akademi University, Turku, Finland). Human primary prostate cancer fibroblasts (CAF) were established as previously described (20), and used up to passage 10. Human primary gingival fibroblasts (GFB) were purchased from ATCC and used up to passage 10. Primary human adult skin fibroblasts (SFB) were from the cell line collection of the Medical Biochemistry/the University of Turku and a kind gift from Professor Risto Penttinen. The fibroblasts were from male donor aged 24 years and they were used up to passage 12. A549, SKOV3, Caov-3, MDA-MB-231, MCF7, Caco-2 and HepG2 cell lines were authenticated by short tandem repeat DNA profiling. Genotyping was performed by the Institute for Molecular Medicine Finland FIMM Technology Centre, University of Helsinki.

All cell lines were grown in Dulbecco’s modified Eagle’s medium (DMEM with 4.5 g/L glucose; 12-614F, Lonza) supplemented with 10% fetal calf serum (FCS), L-glutamine (6 nmol/L), penicillin (100 U/ml) and streptomycin (100 μg/ml). 1 x MEM non-essential amino acids (11140-035, Gibco) were added to UT-SCC-7 and UT-SCC-2 medium. All cell lines were routinely tested to be negative for mycoplasma contamination using MycoAlert PLUS Mycoplasma Detection Kit (LT07-710, Lonza).

### 3D spheroid cultures and hypotonic lysis for mass spectrometric analyses

The information below on spheroid preparation and growth conditions is based on MISpheroID recommendations (21). 3D spheroids were made in micro-molds according to the manufacturer’s instructions (MicroTissues 3D Petri Dish micro-mold spheroids, Sigma-Aldrich) with 2.5 × 10^5^ cells in one mold (monocultures; 7000 cells/spheroid) or 5.0 × 10^5^ cells in one mold (cocultures; 14 000 cells/spheroid). In cocultures the cell ratio was 1:1 (cancer cells and fibroblasts, respectively). The spheroids were grown in serum-free DMEM medium for 5 days at 37°C in an incubator environment of 20% O_2_ and 5% CO_2_. Ascorbic acid (50 µg/ml) was added daily. The spheroids were washed out from the molds with hypotonic lysis buffer (10 mM Tris-HCl pH 7.4, 1 mM EDTA pH 8.0, 10 µg/ml DNase I (DN25, Sigma-Aldrich)) and collected into Eppendorf tubes. The spheroids were then washed twice with hypotonic lysis buffer and centrifuged between washes 2 min/1000 x g. After that, the spheroids were incubated with hypotonic lysis buffer o/n in +4 °C in a rotator. The next day, spheroids were washed twice with hypotonic lysis buffer as described above. The pellets were frozen in -20 °C for further mass spectrometric analysis.

### PAD4 treatment for 3D spheroids

The spheroids were grown in micro-molds for 6 days as described above. Ascorbic acid (50 µg/ml) was added daily. The spheroids were washed out from the molds with citrullination buffer (40 mM Tris-HCl pH 7.4, 5 mM CaCl_2_, 150 mM NaCl) and collected into Eppendorf tubes. The spheroids were washed twice with citrullination buffer and centrifuged between washes 2 min/1000 x g. Next, PAD4 (SAE0086, Sigma-Aldrich; 2 units/sample in citrullination buffer) was added to the spheroids and incubated 2h/+37 °C. Control samples were treated with citrullination buffer only. The spheroids were then washed twice with hypotonic lysis buffer and centrifuged between washes 2 min/1000 x g. After that, the spheroids were incubated with hypotonic lysis buffer o/n in +4 °C in a rotator. The next day, spheroids were washed twice with hypotonic lysis buffer and centrifuged between washes 2 min/1000 x g. The pellets were frozen in -20°C for further mass spectrometric analysis.

### Sample preparation for mass spectrometry

The hypotonically lysed pellets were resuspended in 50 mM triethylammonium bicarbonate (TEAB), 5% SDS, pH 7.5. The dissolved samples were then clarified at 13,000g for 8 minutes. Each sample was digested in an S-Trap micro column (ProtiFi, USA) according to the standard protocol provided by the manufacturer. Briefly, the proteomic mixture in the supernatant was reduced with 20 mM dithiothreitol at 95°C for 20 minutes and then at 60°C for 2 hours. The reduced proteins were alkylated with 40 mM iodoacetamide in dark for 30 minutes. The samples were then treated with 50 mM TEAB, 5% SDS, 7.5 M urea, pH 7.5 to solubilize the matrisome proteins. These ECM solubilized samples were acidified with 1.2% aqueous phosphoric acid and mixed with 6× volumes of 90% methanol, 100 mM TEAB, pH 7.1 (S-Trap binding buffer). A protein solution of 200 µl at a time was loaded onto the filter of the S-Trap micro column and centrifuged at 4000×g for 30 seconds. The flow-through was discarded each time and the samples were washed 9 times with the S-Trap binding buffer. Proteins trapped in the S-Trap column were digested with Trypsin/Lys-C Mix (Promega, USA) at 47°C for 1 hour at a protein-to-enzyme ratio of 1:25 (w/w). The digested peptides were eluted with buffers in order: 50 mM TEAB pH 8.0, 0.2% aqueous formic acid, and 50% acetonitrile/0.2% aqueous formic acid. The eluted fractions were pooled, SpeedVac (Thermo Fisher Scientific, USA) dried, and desalted with C18 tips (Empore, USA). Finally, the desalted peptides were again SpeedVac dried and dissolved in 0.1% formic acid for liquid chromatography-mass spectrometry analysis.

### Liquid chromatography-tandem mass spectrometry

The peptide analysis was performed using a nanoflow HPLC system (Easy-nLC1000, Thermo Fisher Scientific) coupled with a Q Exactive HF mass spectrometer (Thermo Fisher Scientific). The peptide mixture was separated using a 15cm C18 column (75 µm × 15 cm, ReproSil-Pur 3 µm 200 Å C18-AQ, Dr. Maisch HPLC GmbH, Ammerbuch-Entringen, Germany) with a gradient consisting of solvents A (0.1% formic acid) and solvent B (acetonitrile/water (95:5(v/v)) with 0.1% formic acid) from 8–35% B in 50 minutes, 35-100% B in 2 minutes and held at 100% B for 8 minutes. A full mass scan was acquired over the m/z range of 300-1750 at a resolution of 120,000 with a maximum allowed injection time of 50 milliseconds followed by data dependent acquisition of isolation window of 2.0 m/z and dynamic exclusion time of 20 seconds. The top 12 intense peaks from full scan were fragmented with higher-energy collisional induced dissociation and scanned over the m/z range of 200-2000 m/z at a resolution of 15,000. A new survey scan of the full mass spectrum was initiated after the completion of the fragmentation scan, and the process was repeated until the end of the gradient run.

### Data analysis

Raw mass spectrometry data of different cancers and normal tissues were collected from PRIDE (https://www.ebi.ac.uk/pride/), MassIVE (https://massive.ucsd.edu/ProteoSAFe/static/massive.jsp) and CPTAC (https://cptac-data-portal.georgetown.edu/) databases (**Table 1**). All data had been acquired using liquid chromatography-coupled tandem mass spectrometry with data dependent acquisition and QExactive, Orbitrap Lumos or LTQ Orbitrap mass spectrometers.

**Table 1:** Publicly available datasets analyzed in this study.

Sample source^a^	Number of patients^b^	Database	Study ID
Colorectal cancer, patient A ECM	1	MassIVE	MSV0000785555 (22)
Colorectal cancer, patient B ECM	1	MassIVE	MSV0000785555 (22)
Colorectal cancer, patient C ECM	1	MassIVE	MSV0000785555 (22)
Normal colon #1 ECM	1	MassIVE	MSV0000785555 (22)
Normal colon #2 ECM	1	MassIVE	MSV0000785555 (22)
Normal colon #3 ECM	1	MassIVE	MSV0000785555 (22)
Breast cancer ECM	1	PRIDE	PXD005554 (23)
Breast cancer	3	CPTAC	PDC000174 (24)
Colorectal cancer	1	CPTAC	PDC000111 (25, 26)
Hepatocellular carcinoma	5	CPTAC	PDC000198 (27)
Clear cell renal cell carcinoma	5	CPTAC	PDC000127 (28)
Lung adenocarcinoma	4	CPTAC	PDC000153 (29)
Ovarian serous cystadenocarcinoma	3	CPTAC	PDC000113 (30)
Uterine corpus endometrial carcinoma	6	CPTAC	PDC000125 (31)
Liver metastasis of colorectal cancer, patient #1	1	PRIDE	PXD005709 (32)
Liver metastasis of colorectal cancer, patient #2	1	PRIDE	PXD005709 (32)
Liver metastasis of colorectal cancer, patient #3	1	PRIDE	PXD005709 (32)
Liver metastasis of colorectal cancer, patient #4	1	PRIDE	PXD005709 (32)
Liver metastasis of colorectal cancer, patient #5	1	PRIDE	PXD005709 (32)
Liver metastasis of colorectal cancer, patient #6	1	PRIDE	PXD005709 (32)
Liver metastasis of colorectal cancer, patient A ECM	1	MassIVE	MSV0000785555 (22)
Liver metastasis of colorectal cancer, patient B ECM	1	MassIVE	MSV0000785555 (22)
Liver metastasis of colorectal cancer, patient C ECM	1	MassIVE	MSV0000785555 (22)
Lymph node metastasis of melanoma	1	PRIDE	PXD001724 (33)
Plasma (healthy female)	1	PRIDE	PXD007884 (34)
Glioblastoma	10	CPTAC	PDC000204 (35)
Normal brain	1	PRIDE	PXD010154 (36)
Head and neck squamous cell carcinoma	10	CPTAC	PDC000221 (37)
Lung squamous cell carcinoma	5	CPTAC	PDC000234 (38)

a If indicated, the sample has been specifically enriched for ECM proteins.

b TMT or iTRAQ labelled and pooled, if more than one patient. In the case of CPTAC studies, a subset of patient samples was analysed.

Proteome Discoverer 2.4 (Thermo Scientific) with MS Amanda 2.0 search engine (39) was used to analyze all raw data, including in-house generated, against Human reviewed Uniprot sequences including isoforms (release 2020_02). Trypsin-digested peptides with a maximum of 3 missed cleavages were allowed. Parent ion tolerance of 5 ppm and product ion tolerance of 0.02 Da (for data from QExactive or Orbitrap Lumos) or 0.2 Da (for data from LTQ Orbitrap) were allowed. Oxidation (K, M, P) and deamidation (N, Q, R) were set as dynamic modifications. With raw data from TMT or iTRAQ labeled samples, dynamic modification with TMT or iTRAQ label (N-terminus, K) was added. Carbamidomethylation (C) was set as a static modification. Only the peptide spectrum matches (PSMs) with concatenated target-decoy database search-derived false discovery rate below 0.05 were included.

From the potentially citrullinated PSMs, those with C-terminal citrulline were removed, and the spectra matching to matrisome protein-derived peptides according to the human matrisome protein list at http://matrisomeproject.mit.edu (40) were manually inspected and validated. Only the spectra with at least one b or y ion (from collision-induced dissociation) or c or z ion (from electron-transfer dissociation) peak matching to citrullinated peptide fragment with or without neutral loss of isocyanic acid from citrulline residue (-43 Da) were included. Citrullination was determined invalid if (i) citrullination could not be distinguished from N or Q deamidation or (ii) peptide mass had been erroneously calculated from second isotopic peak instead of monoisotopic peak of precursor ion or (iii) all citrullinated peptide fragments could be matched to a second isotopic peak of the same but unmodified fragments or (iv) the retention time was similar or shorter than that of identical unmodified peptide or (v) the citrullinated peptide was the only peptide matching to the corresponding protein or (vi) the peptide contained also unlikely modifications (such as hydroxylation of K or P in non-collagenous proteins). Quantitation of TMT-labeled subset of CPTAC colon adenocarcinoma dataset PDC000116 was done by using MaxQuant, version 1.6.10.43 (41).

### Statistical analysis

Correlation analysis was performed using SPSS Statistics (version 26, IBM). GOnet tool at https://tools.dice-database.org/GOnet/ (42) was used for gene ontology classification.

### Data availability

The mass spectrometry proteomics data generated in this study have been deposited to the ProteomeXchange Consortium *via* the PRIDE (43) partner repository with the dataset identifier PXD033555 and 10.6019/PXD033555. Accession information of publicly available data analyzed in this study can be found in **Table 1**. All other data in this article are available by contacting the corresponding author on reasonable request.

## Results

### Setting the criteria for the detection of citrullinated matrisome proteins by mass spectrometry

**Figure 1:**
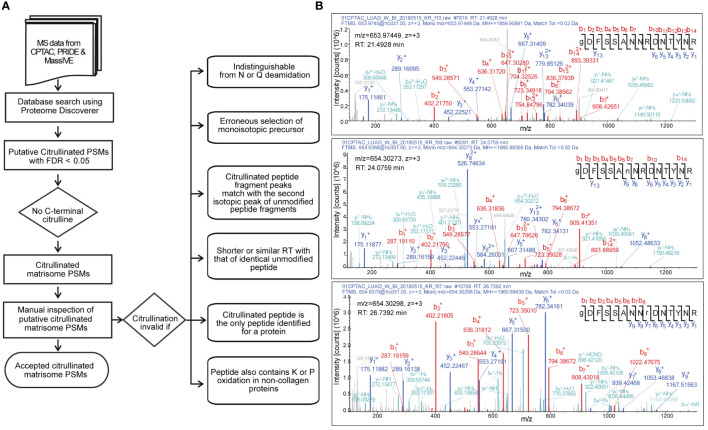
Selection of citrullinated matrisome PSMs. **(A)** A decision tree to accept citrullinated PSMs. **(B)** Example of MS/MS spectra matching to unmodified (top), N-deamidated (middle) and citrullinated (bottom) peptide GDFSSANNRDNTYR of fibrinogen alpha chain from CPTAC lung adenocarcinoma dataset. Although the m/z values of the citrullinated and deamidated precursors are nearly identical, citrullination (r) can be distinguished from deamidation (n). In the bottom spectrum, the existence of y ions 2 to 6 along with b ions 7 and 8 leads to a conclusion that the m/z value of the y7 ion can only be due to citrullination. Retention time (RT) differences with unmodified and deamidated forms give even further evidence for citrullination.

Citrullination of an arginine residue causes 0.98 Da mass increase, which can be detected by mass spectrometry. However, the risk to falsely interpret a peptide as citrullinated is high. The mass shift caused by citrullination is identical to that of spontaneous deamidation of asparagine or glutamine. Thus, it is crucial to include deamidation of asparagine and glutamine along with citrullination as variable modifications in the database search. We also included oxidation of proline and lysine as variable modifications, since we were specifically interested in matrisome proteins, including collagens. We used several criteria for rejecting the spectra matching to citrullinated peptides (**Figure 1**).

After initial database search and selection of peptide-spectrum matches (PSMs) with false discovery rate less than 0.01, we categorically rejected the PSMs with C-terminal citrullination. It has been shown that trypsin is about 10^5^ times slower in hydrolyzing benzoyl-L-citrulline-methyl ester than benzoyl-L-arginine methyl ester (44). Therefore, it is reasonable to assume that citrullination leads to missed cleavage, and thus the presence of citrulline residue in the peptide C- terminus would be possible only in C-terminal peptides derived from proteins with C-terminal arginine.

Since our focus was in matrisome proteins, we included only the spectra matching to proteins listed in the matrisome database (http://matrisomeproject.mit.edu). The relatively low number of the remaining spectra allowed us to carefully examine them manually. Citrullination was determined invalid if it could be confused with deamidation of asparagine or glutamine. Moreover, since the mass shift of one citrullination (0.98 Da) is close to mass of neutron (1.01 Da), the search program occasionally determined the second isotopic peak as monoisotopic peak, leading to false identification of citrullinated peptide. We also noticed that the fragmentation spectra contained second (and third) isotopic peaks of the b and y ions, and occasionally the search program interpreted the second isotopic peak as a monoisotopic peak, leading to falsely detected citrullination. This together with erroneous determination of monoisotopic precursor peak or confusing with deamidation could result in false identification of citrullinated peptide by the database search.

Citrullination increases hydrophobicity of the peptide leading to longer retention time compared to the identical unmodified peptide. Moreover, deamidation of asparagine or glutamine causes smaller shift of retention time compared to that of citrullination (45). Thus, we determined citrullination invalid if the retention time of the identical unmodified peptide was similar or shorter.

It is a common practice and recommended by *e.g.,* in the guidelines of Molecular and Cellular Proteomics journal (https://www.mcponline.org/mass-spec-guidelines) in mass spectrometry-based proteomics that a protein is determined as identified only if it has at least two peptide identifications. Therefore, we did not accept spectra that matched to a protein with no other peptide identifications. In addition, we rejected the spectra that matched to a peptide containing unlikely modifications such as proline hydroxylation in a non-collagenous protein.

Fragmentation by collision-induced dissociation causes neutral loss of isocyanic acid (43 Da) from citrullinated peptide. Although peaks representing y and b ions with 43 Da loss can be frequently seen in the fragmentation spectra of citrullinated peptides, this is not always the case (45). Therefore, we decided not to include the absence of neutral loss of 43 Da as a criterion of falsely determined citrullination.

### Citrullination of matrisome proteins can occasionally be detected in human tumors

**Figure 2:**
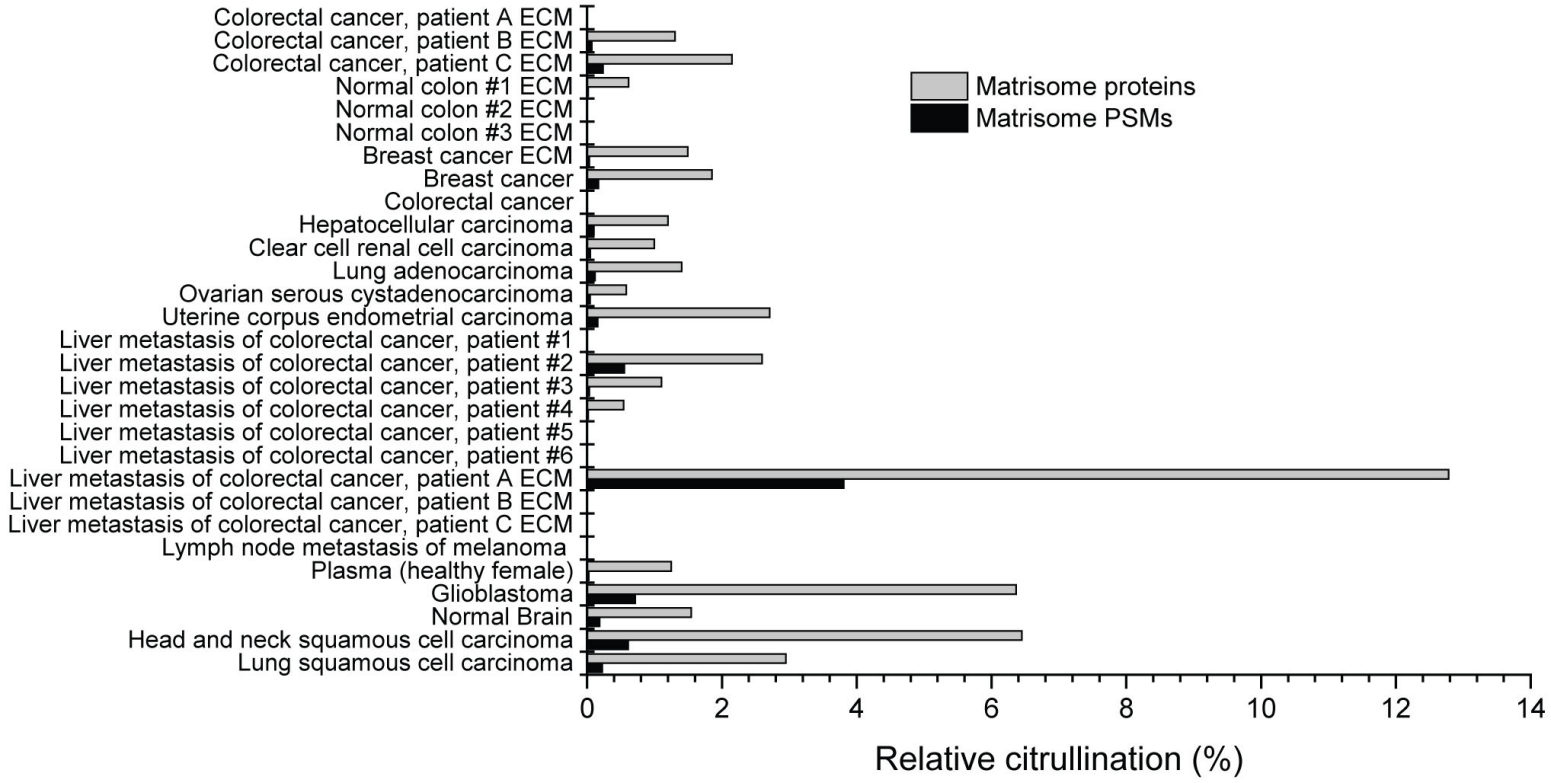
Citrullination of matrisome in various cancer and normal tissue samples. Raw data was obtained from public repositories (**Table 1**) and searched against Human Uniprot sequences including isoforms. The results were filtered to include only the citrullinated spectra matching to matrisome proteins. Only those citrullinated matrisome PSMs were accepted that passed the criteria presented in **Figure 1**. Relative citrullination was calculated by dividing the number of citrullinated matrisome PSMs/proteins with all detected matrisome PSMs/proteins in the sample. Matrisome protein was defined as citrullinated if it had at least one citrullinated PSM. As indicated, some datasets are from samples specifically enriched for ECM proteins.

To study the abundance of ECM citrullination in human tumors, we analyzed cancer proteomics data sets in PRIDE, MassIVE and CPTAC (**Supplementary Tables 1, 2**) for citrullination of matrisome proteins. The data sets contained both pooled cancer samples and samples derived from individual patients (**Table 1**). Citrullinated matrisome proteins were detected in 16 out of 24 cancer related data sets and in 3 out of 5 data sets of normal tissue samples. However, only in three data sets the relative citrullination of matrisome proteins was higher than 4%. These data sets represented a liver metastasis from one patient with a colorectal cancer, CPTAC glioblastoma and CPTAC head and neck squamous cell carcinoma samples (**Figure 2**). Thus, the conclusion was that although small numbers of citrullinated matrisome proteins can be detected in many tumors, it is a relative rare phenomenon. Still some tumors may contain higher numbers of citrullinated matrisome proteins, and in these cases, it is also possible to speculate that citrullination contributes to pathogenic mechanisms. The most often citrullinated matrisome protein was fibrinogen alpha chain (FGA; **Table 2**). Citrullinated peptides derived from FGA were found in 14 out of 29 data sets (**Table 2**). Previously, citrullination of fibrinogen has been connected to inflammatory diseases, such as rheumatoid arthritis (11, 46). Other most frequently citrullinated matrisome proteins included coagulation factor II (F2, thrombin; 9/29 data sets), cathepsin G (CTSG), fibronectin 1 (FN1), and periostin (POSTN, osteoblast specific factor) (7/29 data sets; **Table 2**). Citrullination of fibronectin is also an abundant phenomenon in inflammation (11, 46–48).

**Table 2:** Most frequently citrullinated matrisome proteins.

Gene Symbol	Protein name	Uniprot ID	Division^a^	Category^a^	Citrullination frequency^b^
FGA	fibrinogen alpha chain	P02671	Core matrisome	ECM Glycoproteins	14
F2	coagulation factor II (thrombin)	P00734	Matrisome-associated	ECM Regulators	9
CTSG	cathepsin G	P08311	Matrisome-associated	ECM Regulators	7
FN1	fibronectin 1	P02751	Core matrisome	ECM Glycoproteins	7
POSTN	periostin, osteoblast specific factor	Q15063	Core matrisome	ECM Glycoproteins	7
KNG1	kininogen 1	P01042	Matrisome-associated	ECM Regulators	6
FGB	fibrinogen beta chain	P02675	Core matrisome	ECM Glycoproteins	5
ITIH4	inter-alpha (globulin) inhibitor H4	Q14624	Matrisome-associated	ECM Regulators	5
VTN	vitronectin	P04004	Core matrisome	ECM Glycoproteins	5
HSPG2	heparan sulfate proteoglycan 2	P98160	Core matrisome	Proteoglycans	4
PLG	plasminogen	P00747	Matrisome-associated	ECM Regulators	4
A2M	alpha-2-macroglobulin	P01023	Matrisome-associated	ECM Regulators	3
AMBP	alpha-1-microglobulin/bikunin precursor	P02760	Matrisome-associated	ECM Regulators	3
ANXA6	annexin A6	P08133	Matrisome-associated	ECM-affiliated Proteins	3
COL6A3	collagen, type VI, alpha 3	P12111	Core matrisome	Collagens	3
ELANE	elastase, neutrophil expressed	P08246	Matrisome-associated	ECM Regulators	3
ITIH2	inter-alpha (globulin) inhibitor H2	P19823	Matrisome-associated	ECM Regulators	3
VWA3B	von Willebrand factor A domain containing 3B	Q502W6	Core matrisome	ECM Glycoproteins	3

a Classification according to the Matrisome Project (http://matrisomeproject.mit.edu).

b Number of datasets (total of 29) containing spectra matching to citrullinated peptide(s).

### Cancer cells do not promote citrullination of matrisome proteins in spheroid cell cultures

Our analysis of cancer proteomics data sets revealed that some malignant tumors seem to contain significant numbers of citrullinated matrisome proteins. However, it is unclear which cells in the tumor are required for citrullination. To investigate this, we used three-dimensional spheroid-type cell cultures that are widely used in *in vitro* studies focused on stromal interactions in cancer (49). When cancer cells are cocultured with fibroblasts the basic composition of ECM is quite similar as in the corresponding tumors (20). In a spheroid most of the ECM proteins are produced by fibroblasts, whereas cancer cells have usually a minor contribution only (20). Here we used eight different human cancer cell lines, namely SKOV3 and Caov3 (ovarian adenocarcinoma), A549 (lung carcinoma), MDA-MB-231 and MCF7 (breast adenocarcinoma), UT-SCC2 (head and neck squamous cell carcinoma), Caco2 (colon adenocarcinoma), and HepG2 (hepatocellular carcinoma), which were cocultured (1:1) with human cancer associated fibroblasts (CAF). Spheroids were grown for 5 days in serum-free medium. Ascorbic acid (50 µg/ml) was added daily to allow the synthesis of proper, hydroxyproline and hydroxylysine containing collagen. After lysis of the spheroids, the proteins in the insoluble fraction were isolated and digested, and the resulting peptides were analyzed by mass spectrometry. We could not recognize any citrullinated peptides derived from ECM proteins (**Table 3**). Thus, we concluded that factors derived from cancer cells or fibroblasts may not be responsible for extracellular citrullination in tumors.

**Table 3:** Number of spectra matching to citrullinated or to all peptides obtained from the mass spectrometric analysis of spheroid cocultures of cancer cell lines and CAFs.

Spheroid culture	All PSMs	Citrullinated PSMs	Matrisome PSMs	Citrullinated matrisome PSMs
SKOV3 + CAF	23766	71	1808	0
A549 + CAF	23324	41	775	0
MDA-MB-231 + CAF	22668	35	1874	0
Caov3 + CAF	20869	43	849	0
UT-SCC-2 + CAF	19742	28	1231	0
CaCo2 + CAF	24531	35	898	0
MCF7 + CAF	23963	66	1714	0
HepG2 + CAF	23596	47	2276	0
CAF	21913	31	1978	0

In another set of experiments, we used a ninth cancer cell line, UT-SCC-7 cutaneous squamous cell carcinoma cells, together with human skin fibroblasts (SFB) and UT-SCC-2 cells together with gingival fibroblasts (GFB) in spheroid cocultures. Spheroids were grown for 6 days in serum-free medium. Ascorbic acid was added daily. When ECM protein related peptides were analyzed by mass spectrometry, we did not identify any citrullinated peptide derived from matrisome proteins (**Table 4**).

**Table 4:** Number of spectra matching to citrullinated or to all peptides obtained from the mass spectrometric analysis of spheroid co-cultures of cutaneous squamous cancer cell lines and human fibroblasts with or without PAD4 treatment before lysis.

Spheroid culture	Treatment	All PSMs	Citrullinated PSMs	Matrisome PSMs	Citrullinated matrisome PSMs
GFB	Control	11025	14	1119	0
GFB	PAD4	17144	88	1382	0
GFB + UT-SCC-2	Control	25374	15	1217	0
GFB + UT-SCC-2	PAD4	23394	110	1155	0
SFB	Control	14429	11	1903	0
SFB	PAD4	16220	20	1902	0
SFB + UT-SCC-7	Control	23553	18	1681	0
SFB + UT-SCC-7	PAD4	24726	87	2009	1

We also treated the spheroids with PAD4 to enzymatically citrullinate ECM proteins *in vitro*. We could not detect any citrullinated matrisome-derived peptides after PAD4 treatment, except for one peptide derived from fibroblast growth factor binding protein 1 (FGFBP1), which is not an integral part of ECM (**Table 4**). We could detect from two- to seven-fold increase in the number of spectra matching to citrullinated non-matrisome-derived peptides after enzymatic citrullination (**Table 4**), which indicates that PAD4 had functioned as expected. Classification of the citrullinated proteins in PAD4-treated spheroids by selected cellular compartment (CC) gene ontology terms using GOnet Tool (https://tools.dice-database.org/GOnet/) revealed that most of the citrullinated non-matrisome derived peptides in PAD4-treated spheroids were intracellular (**Supplementary Figure S1**), which suggests that externally added PAD4 had citrullinated proteins derived from degraded or necrotic cells, which are common in spheroid cultures. The fact that externally added PAD4 could not citrullinate matrisome proteins in spheroids is in disagreement with the fact that PADs can citrullinate *e.g.*, structural ECM proteins in chronic inflammation. However, at the site of inflammation ECM is often intensively degraded by proteolytic enzymes, such as matrix metalloproteinases (MMPs), and the intact matrix in spheroids may protect matrisome proteins against PAD4.

### Citrullination of matrisome proteins in tumors correlates with the presence of inflammatory cells

**Figure 3:**
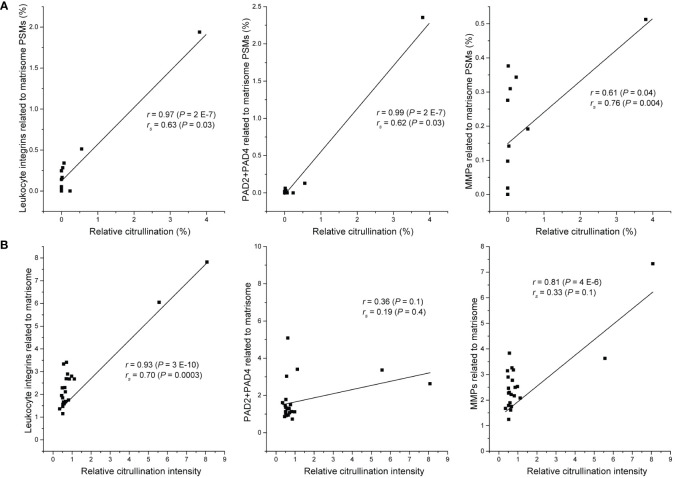
Relation between citrullination of matrisome and selected inflammation markers. **(A)** Metastatic and tumor samples of colorectal cancer patients (20). Relative citrullination was calculated by dividing the number of citrullinated matrisome PSMs with all detected matrisome PSMs in the sample (n = 12). Sum of PSMs of PAD2 and PAD4, all detected matrix metalloproteins (MMPs) or all detected leukocyte integrins (ITGAM, ITGAX, ITGB2) was divided by the number of all matrisome PSMs in the sample. **(B)** Subset of CPTAC colon adenocarcinoma dataset containing only tumor tissue samples (n = 22). Relative citrullination intensity was calculated by dividing median intensity of citrullinated PSMs with median intensity of matrisome PSMs in the sample. Median intensity of PSMs of PAD2 and PAD4, all detected matrix metalloproteins (MMPs) or all detected leukocyte integrins (ITGAM, ITGAX, ITGB2) was divided by the median intensity of all matrisome PSMs in the sample. r, Pearson correlation coefficient; r_s_, Spearman correlation coefficient; P, two-tailed P-value.

Our observations based on spheroid cell cultures failed to find any evidence that cancer cells or stromal fibroblasts could actively citrullinate matrisome proteins. Previously, citrullination of extracellular proteins has been well described during chronic inflammation (9). Inflammatory cells are also frequently found in malignant tumors. Furthermore, some of the proteins that we found to be most frequently citrullinated in cancer data sets, *e.g.*, fibrinogen and fibronectin, are often citrullinated in inflammation (11, 46). Thus, it is reasonable to speculate that the citrullination of matrisome proteins in cancer may also be due to PADs released by inflammatory cells. To test this hypothesis in the case of colorectal cancer that has been suggested to be dependent on citrullination for metastatic growth (4), we selected only the experiments where several primary or metastatic colorectal cancer samples had been analyzed individually (22). The data from these samples contained spectra matching to inflammation-related proteins, such as PAD enzymes, MMPs (MMP1, MMP2, MMP7, MMP8, MMP9, MMP11, MMP12, MMP14, MMP19 and MMP23) and also inflammatory cell-specific receptors, namely leukocyte integrins. We found a statistically significant correlation between citrullinated matrisome PSMs and PAD2 + PAD4 PSMs (p=0.03), between citrullinated matrisome PSMs and MMP PSMs (p=0.004) and between citrullinated matrisome PSMs and leukocyte integrin PSMs (p=0.03) (**Figure 3A**). Specifically, it appears that an exceptionally high citrullination level is connected to high level of inflammation markers.

To confirm that the relation between citrullination and inflammation markers is not restricted to only one dataset, we analyzed a subset of tumor tissue samples (n = 22) of CPTAC colon adenocarcinoma dataset PDC000116 (50) for citrullination and used MaxQuant software (41) to quantitate the TMT labelled samples. In this subset, the samples with high citrullination level had also high level of leukocyte integrins and MMPs (**Figure 3B**). There seemed to be no clear relation between citrullination and PADs, which may be explained by the fact that, unlike the samples in **Figure 3A**, CPTAC samples have not been enriched for ECM and thus contain also intracellular PADs. Nevertheless, these results suggest that in malignant tumors citrullination of matrisome proteins is associated to inflammation-related release of PADs and matrix degradation rather than direct action of cancer cells or stromal fibroblasts. Furthermore, the level of citrullination varies substantially between metastasis samples of colorectal cancer (**Figure 2**), which further support the idea that citrullination is a side effect of inflammation rather than a prerequisite for metastatic growth.

## Discussion

Post-translational modifications (PTMs), such as citrullination, have the potency to increase the structural and functional diversity of ECM proteins and fibrils (51). Furthermore, autoantibodies against citrullinated epitopes in proteins, including matrisome proteins, are a hallmark of rheumatoid disease (52). These antibodies may sustain inflammation and target it to ECM-rich tissues, *e.g.*, cartilage (52). In tumors, transformed cancer cells actively interact with many other cell types and ECM, but the putative citrullination of matrisome proteins in human cancer has generated much less interest than in inflammation. The new analysis of cancer proteomics data sets in PRIDE, MassIVE and CPTAC indicates that large-scale citrullination of matrisome proteins in tumors is not a common phenomenon. Still, in some data sets it is possible to find citrullinated matrisome PSMs suggesting that in these cases protein citrullination may be a source of neoantigens (17).

Previously, citrullination of matrisome proteins has been reported in metastatic colon cancer (4). Detection of citrullinated PSMs by mass spectrometry has several pitfalls and it is critical to check what criteria have been used when citrullination has been analyzed. The most obvious criteria are the spectrum quality, correct monoisotopic ion determination and distinction from deamidation of N or Q, and we have included these to our criteria, too. Yuzhalin et al. (4) used an unrestricted modification search strategy where all theoretical modifications are searched simultaneously (53). However, since the frequency of false positives increases with the number of modifications searched (54), using this strategy can potentially increase the probability of false identification of citrullinated peptide and result in erroneous detection of citrullinated peptides with unlikely additional modifications. Therefore, here we have used only oxidation of M (common spontaneous modification), deamidation of N and Q (common spontaneous modification with the same mass shift as that of citrullination) and hydroxylation of P, and K (common modifications in collagenous proteins) along with citrullination as variable modifications in the database search. In addition, given that the number of spectra matching to a citrullinated peptide is considerably lower than the number of spectra matching to an unmodified peptide of the same protein, we have rejected all spectra matching to a citrullinated peptide with unlikely additional modifications or to a protein having no other evidence of identification. We have also exploited the fact that citrullination increases hydrophobicity leading to a longer retention time (4245) and rejected the citrullinated PSMs that have similar or shorter retention time as their non-citrullinated counterparts. Lee et al. (55), who conducted a large-scale mining of deep proteomic data of 30 human tissues, used also the neutral loss of isocyanic acid as a criterion of citrullination. However, we decided not to include it to our criteria, since the peak of this ion is not always visible in tandem mass spectra of citrullinated peptides (45).

PAD enzymes are expressed in cancer cells, and their main substrates are intracellular proteins. The mechanism of citrullination of extracellular proteins by PADs is not known very well. It is possible that cells have some mechanism to release PADs to interstitial space, either directly or in exosomes or extracellular vesicles (56, 57). In cancer, cell death and degradation are also quite common mechanisms that may explain the interaction of extracellular proteins with intracellular enzymes. Inflammatory cells are also known to release PADs. One possible mechanism is related to neutrophil extracellular traps (NETs). NETs are web-like structures of chromatin decorated with a variety of other proteins. PADs have been connected to the formation of NETs that are released by neutrophils in a process called NETosis (58). Interestingly, NETs have been detected in *e.g.*, colon cancer and they may participate in processes such as mesenchymal-epithelial transition (59). Our conclusion that the citrullination of matrisome proteins in cancer is mainly related to inflammation is based on the following facts: Firstly, analysis of cancer proteomics data sets revealed that many of the most frequently citrullinated matrisome proteins, such as fibrinogen and fibronectin, are also abundantly citrullinated in *e.g.*, synovial fluid in rheumatoid disease (11). Secondly, we could not detect cancer cell or fibroblast associated citrullination in a three-dimensional spheroid cell culture system that in many ways mimics tumor microenvironment (60). Finally, citrullination of matrisome proteins in tumors significantly correlated with the presence of leukocyte specific receptors.

In our cell culture experiments the treatment of spheroids with PAD4 led to the increased citrullination of intracellular proteins. The fact that spheroids also contain dead and dying cells may explain this phenomenon, since the intracellular proteins released after cell death are susceptible for citrullination by externally added PAD4. Furthermore, the result suggests that PADs released by inflammatory cells may also citrullinate proteins of intracellular origin. Importantly, the citrullinated forms of intracellular proteins, such as α-enolase (61) and nucleophosmin (62) are potential targets in cancer immunotherapy.

Citrullination of matrisome proteins may also affect their function. Arginine residues are often located at the critical protein–protein interaction sites (11). A good example is that integrin family cell adhesion receptors can recognize motifs such as RGD in fibronectin and fibrinogen and triple helical GFOGER (O=hydroxyproline) in collagens. Integrins mediate the anchorage of cells to ECM and orchestrate chemical and mechanical signal transduction that may play critical role in the regulation of cancer cell behavior. Interestingly, we could detect citrullination of an additional integrin ligand, periostin. In cancer periostin has been associated with the regulation of cell survival, invasion, angiogenesis, metastasis, and the epithelial-mesenchymal transition (63, 64). We also identified citrullinated peptides of basement membrane-specific heparan sulfate proteoglycan core protein (HSPG2). Angiostatic activity of endorepellin, which is a C-terminal module of HSPG2, has been shown to be mediated by integrin interaction (65).

To conclude, our results indicate that in human cancer, inflammation promotes citrullination of matrisome proteins. Citrullination has the potency to affect protein function and create neoantigens, and consequently modify the cancer-related cellular processes.

## Data availability statement

The datasets presented in this study can be found in online repositories. The names of the repository/repositories and accession number(s) can be found below: https://www.ebi.ac.uk/pride/archive/, PXD033555.

## Author contributions

JH conceived the project. JH, PR, US and ES planned the experiments. ES performed the cell experiments. PR and US conducted mass spectrometry experiments and data analysis. JH, PR, and US wrote the manuscript. All authors contributed to manuscript preparation. The work reported in the paper has been performed by the authors, unless clearly specified in the text.

## Funding

This research was supported by the grants from Jane and Aatos Erkko Foundation, Sigrid Jusélius Foundation, Finnish Cancer Research Foundation and the Academy of Finland (grant 329743).

## Acknowledgments

Mass spectrometry analyses were performed at the Turku Proteomics Facility, University of Turku, supported by Biocenter Finland. We thank the Core Facilities of the Turku Bioscience Centre. Maria Tuominen is acknowledged for skillful technical assistance.

## Conflict of interest

The authors declare that the research was conducted in the absence of any commercial or financial relationships that could be construed as a potential conflict of interest.

## Publisher’s note

All claims expressed in this article are solely those of the authors and do not necessarily represent those of their affiliated organizations, or those of the publisher, the editors and the reviewers. Any product that may be evaluated in this article, or claim that may be made by its manufacturer, is not guaranteed or endorsed by the publisher.

## Abbreviations

CAF, cancer-associated fibroblast; DMEM, Dulbecco’s modified Eagle’s medium; ECM, extracellular matrix; GFB, gingival fibroblast; MMP, matrix metalloproteinase; PAD, protein arginine deiminase; PSM, peptide-spectrum match; SCC, squamous cell carcinoma; SFB, skin fibroblast; TEAB, triethylammonium bicarbonate.
