# Supplementary figures and images for: Inflammation-related citrullination of matrisome proteins in human cancer
Source: Front Oncol. 2022 Dec 1;12:1035188. doi: 10.3389/fonc.2022.1035188 (PMC9753687; doi:10.3389/fonc.2022.1035188)

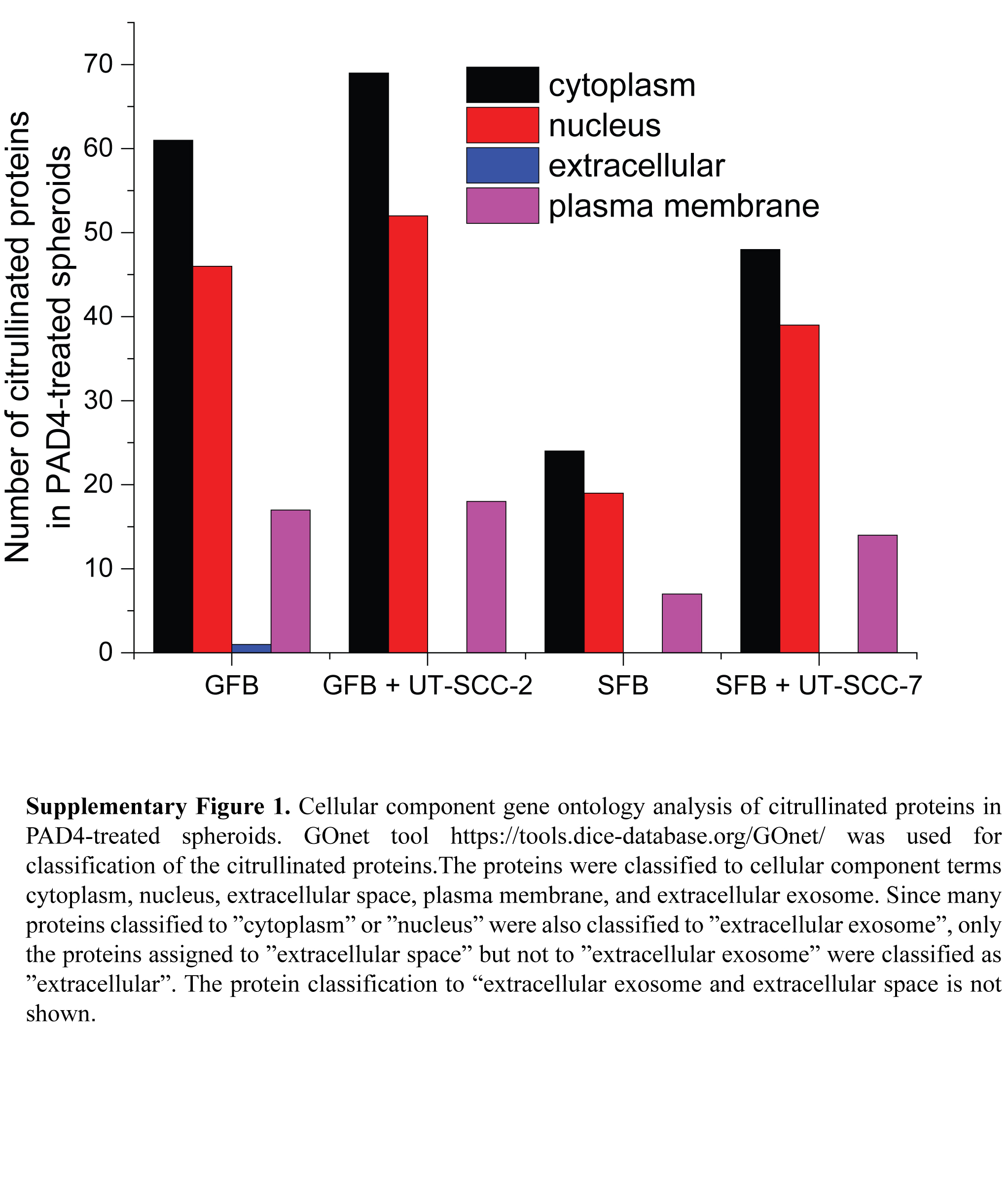

Supplement: Supplementary file 1 [file Image_1.jpeg]
